# Serum Mannose-Binding Lectin Concentration, but Not Genotype, Is Associated With *Clostridium difficile* Infection Recurrence: A Prospective Cohort Study

**DOI:** 10.1093/cid/ciu666

**Published:** 2014-08-28

**Authors:** Andrew Swale, Fabio Miyajima, Ruwanthi Kolamunnage-Dona, Paul Roberts, Margaret Little, Nicholas J. Beeching, Mike B. J. Beadsworth, Triantafillos Liloglou, Munir Pirmohamed

**Affiliations:** 1 The Wolfson Centre for Personalised Medicine, University of Liverpool; 2 The Royal Liverpool and Broadgreen University Hospitals NHS Trust; 3 Department of Biostatistics, University of Liverpool; 4 Liverpool School of Tropical Medicine; 5 Health Protection Unit in Gastrointestinal Infections, National Institute for Health Research; 6 Cancer Research Centre, University of Liverpool, United Kingdom

**Keywords:** *Clostridium difficile*, CDI, MBL, disease recurrence

## Abstract

***Background.*** Mannose-binding lectin (MBL) plays a key role in the activation of the lectin-complement pathway of innate immunity, and its deficiency has been linked with several acute infections. However, its role in predisposing to, or modulating disease severity in, *Clostridium difficile* infection (CDI) has not been investigated.

***Methods.*** We prospectively recruited 308 CDI case patients and 145 control patients with antibiotic-associated diarrhea (AAD). CDI outcome measures were disease severity, duration of symptoms, 30-day mortality, and 90-day recurrence. Serum concentrations of MBL were determined using a commercial enzyme-linked immunosorbent assay transferred to an electrochemiluminescence–based platform. *MBL2* polymorphisms were typed using a combination of pyrosequencing and TaqMan genotyping assays.

***Results.*** The frequency of the *MBL2* genetic variants was similar to that reported in other white populations. MBL serum concentrations in CDI and AAD subjects were determined by *MBL2* exonic variants *B*, *C*, and *D* and the haplotypes (*LYPB*, *LYQC*, and *HYPD*). There was no difference in either MBL concentrations or genotypes between cases and controls. MBL concentration, but not genotype, was a determinant of CDI recurrence (odds ratios, 3.18 [95% confidence interval {CI}, 1.40–7.24] and 2.61 [95% CI, 1.35–5.04] at the <50 ng/mL and <100 ng/mL cutoff points, respectively; *P* < .001). However, neither MBL concentration nor *MBL2* genotype was linked with the other CDI outcomes.

***Conclusions.*** Serum MBL concentration did not differentiate between CDI cases and AAD controls, but among CDI cases, MBL concentration, but not genotype, was associated with CDI recurrence, indicating that MBL acts as a modulator of disease, rather than a predisposing factor.

## Abstract (precis)

Low mannose-binding lectin concentration, but not genotype, was associated with disease recurrence in a large prospective cohort of patients with *Clostridium difficile* infection.

The initiation and propagation of inflammatory cascades is an essential housekeeping property of the innate immune response during infections. Mannose-binding lectin (MBL) activates the lectin-complement pathway of innate immunity through binding to repetitive sugar arrays on microbial surfaces [1]. MBL is also a potent regulator of inflammatory pathways: it can modulate phagocyte interaction with mucosal organisms at the site of infection [2], and interacts with other components of the innate immune system such as Toll-like receptors [3].

Low MBL concentrations have been associated with increased susceptibility to infections in both animal models and humans [4, 5], as well as with poor disease prognosis [1]. The modulation of disease severity is partly thought to be through a complex, dose-dependent influence on cytokine production [6]. Serum MBL concentrations range from negligible to as high as 10 000 ng/mL [7–9]; this varies with ethnicity and with the screening method adopted [10].

MBL secretion in humans is dependent on the *MBL2* genetic architecture [11, 12]. To date, 57 genetic variants have been identified within the entire *MBL2* gene (dbSNP, build 140, NCBI), with only 6 of them known to affect secretion and/or function of the encoded protein (Figure 1) [8, 13]. The mutated alleles *B*, *C*, or *D* are collectively termed *O* and their correspondent wild-type alleles are jointly referred to as variant *A*, with the presence of any given *O* variant (in either the heterozygous or homozygous state) resulting in MBL deficiency [8, 13]. The existence of strong linkage disequilibrium (LD) between the promoter and structural gene variants means that only 7 common haplotypes (out of a possible 64), which may affect serum concentrations, have been described: *HYPA*, *LYQA*, *LYPA*, *LXPA*, *HYPD*, *LYPB*, and *LYQC* [14, 15]. *HYPD*, *LYPB*, and *LYQC* lead to the production of unstable ligands with shorter half-lives that are easily degraded to lower oligomeric forms. Studies that have evaluated both genetic mutations and serum concentrations in white populations are summarized in Supplementary Table 1.

**Figure 1.**
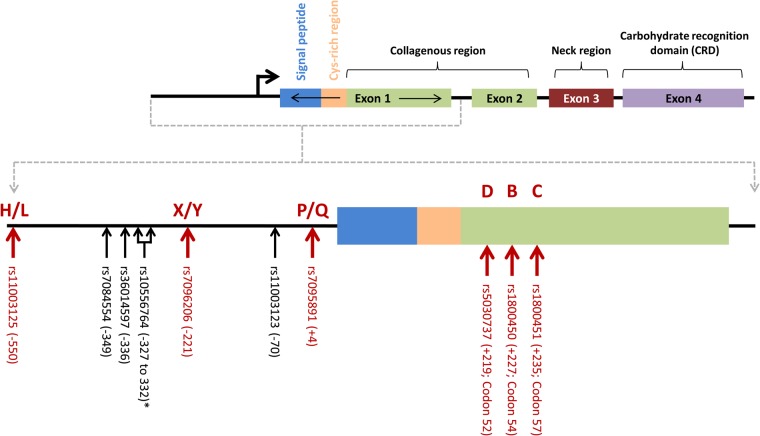
Schematic representation of the major *MBL2* isoform and genetic polymorphisms. Polymorphisms responsible for the haplotypes that ultimately determine mannose-binding lectin (MBL) expression levels are indicated by the red arrows. *In this study, rs10556764 (6-bp deletion) was used as a proxy single-nucleotide polymorphism for rs7095891.

*Clostridium difficile* is an opportunistic spore-forming bacterium that can effectively colonize the intestinal tract following antibiotic-driven dysbiosis. *Clostridium difficile* infection (CDI) is the result of intense colonic inflammation caused by the release of potent enterotoxins. Research into host biomarkers for CDI has focused on mediators of inflammation in the gut, such as fecal interleukin 8 [16], lactoferrin [16], and calprotectin [17], and linked them with disease severity [16, 18]. More recently, both serum interleukin 23 and procalcitonin have also been proposed as potential biomarkers for CDI severity [19, 20]. However, the role of these biomarkers in the stratification of problematic CDI patients remains unclear, and thus remains an important area of research. Additionally, several clinical prediction rules have been proposed for the evaluation of CDI outcomes [21–23], but none have gained widespread clinical acceptance.

To date, there have been no studies on the role of either MBL levels or *MBL2* genetic variants with CDI, possibly because MBL is not thought to bind to the surface of *C. difficile* [24]. However, there is growing evidence for an association between MBL and major modulators of inflammation, such as Toll-like receptors and C-reactive protein (CRP), both of which have been associated with CDI [25, 26]. Therefore, we sought to investigate the role of MBL in a prospective cohort of CDI cases and inpatient controls.

## METHODS

### Cohort

A cohort of 453 inpatients was consecutively recruited from a large hospital setting in Merseyside, United Kingdom. Patients were eligible for inclusion if they had healthcare-associated diarrhea (defined as ≥3 liquid stools passed in the 24 hours preceding assessment), an onset after being in hospital for >48 hours, and recent exposure to either antimicrobials and/or proton pump inhibitors (PPIs). Using criteria previously described [27], 308 patients with CDI (cases) and 145 control patients with antibiotic-associated diarrhea (AAD) were classified based on toxin enzyme-linked immunosorbent assay (ELISA) test (TOX A/B II, Techlab, Blacksburg, Virginia), microbiological culture, and clinical diagnosis made by independent clinicians. Polymerase chain reaction (PCR) ribotyping and multiplex PCR were performed to determine strains types and the toxigenic nature of the isolates [28].

Blood and fecal specimens were collected from patients at study entry, of whom 98% were white. Relevant information on demographics, admission, and clinical history was collected for each patient. Ethical approval was obtained from the Liverpool Research Ethics Committee (reference number 08/H1005/32), and each patient provided written informed consent prior to recruitment.

### Definition of Outcomes

Cases and controls were defined as described above. The severity of CDI symptoms was assessed at baseline by research nurses using the guidelines proposed by Public Health England [29], which we adjusted to incorporate a more stringent white blood cell count cutoff of >20 × 10^9^/L while also replacing acute rising creatinine with an estimated glomerular filtration rate of <30 mL/min/1.73 m^2^ at the time of diagnosis. Duration of symptoms was recorded from the date of onset of symptoms and then dichotomized into episodes lasting ≥10 or <10 days. All-cause mortality was actively monitored for a period of 30 days from diagnosis, and recurrent CDI was defined as the development of subsequent CDI episodes up to a period of 90 days post-diagnosis of the initial episode. If the patient was discharged from hospital prior to final follow-up, we attempted in every case to obtain data from the hospital, general practitioner, or patient (the latter by a telephone call).

### Determination of MBL Serum Concentrations

A commercially available in vitro diagnostic ELISA kit (Sanquin Blood Supply, Amsterdam, the Netherlands) was transferred onto the Meso Scale Discovery electrochemiluminescence (ECL)–based platform, undergoing appropriate optimization prior to use. The MBL kit control was used across all plates to determine interplate variability and a subsequent correction factor used for each plate. Final minimum detection level (lower limit of detection [LLOD]) and minimum quantification level (lower limit of quantification [LLOQ]) were calculated by taking the mean values across all plates. The mean LLOD and LLOQ across all plates were 11.3 and 11.0 ng/mL, respectively, with overall median values of 491.9 ng/mL among controls and 361.8 ng/mL in cases. Signal values ranged from only 50 to 500 ECL units, which denotes a compressed signal range inherent with the assay. Because this may have potentially limited discrimination of the quantitative values, data were subject to binary categorization based on 3 previously used deficiency cutoffs: 50, 100, and 500 ng/mL [30–32].

### Determination of *MBL2* Variants

A total of 9 variants lying in the promoter and exon 1 were typed (Figure 1) by either pyrosequencing (PyroMark Q96 custom assays, Qiagen; rs36014597, rs7084554, rs1800451, rs1800450, rs5030737, and rs10556764) or TaqMan SNP genotyping (Applied Biosystems; rs7096206, rs11003125, and rs11003123). The variants rs1800451 (*C*), rs1800450 (*B*), rs5030737 (*D*), rs7096206 (*X/Y*), and rs11003125 (*H/L*) were used for haplotype determination, and rs10556764, a 6-bp Ins/Del in complete LD with rs7095891 (*P/Q*), was used as a proxy*.* Another recognized tagging marker for *P/Q* (rs11003123) was independently typed to evaluate the accuracy of the pyrosequencing assays.

#### Pyrosequencing

PCR optimization was conducted using 20 ng of genomic DNA and temperature gradients following standard guidelines. Optimized products were run on a PyroMark Q96 ID following the recommended assay protocol. Repeat samples and blanks were included for quality control purposes, and data were analyzed using PyroMark Q96 software (version 2.5.8).

#### TaqMan Genotyping

Reactions consisted of 20 ng genomic DNA, 1× TaqMan SNP genotyping assays, run on an Applied Biosystems HT 7900 Fast Real-time PCR system (Applied Biosystems) using standard cycling conditions. Repeat samples and blanks were incorporated for quality control purposes, and results analyzed using SDS software (version 2.2).

### Statistical Analysis

Median MBL serum concentrations were compared for individual SNPs and haplotypes by the Mann–Whitney *U* test, then subjected to stratification based upon previously used 2-marker grouping profiles termed high- (*YA/YA* and *XA/YA*), intermediate- (*XA/XA* and *YA/YO*), and low-expressing (*XA/YO* & *YO/YO*) genotypes [32, 33].

The effect of both *MBL2* genetics (based on stratified expression genotypes) and serum MBL concentrations (based upon deficiency cutoffs) were individually taken forward for case-control comparison and subgroup analysis of cases. For the latter, this included logistic regression for the following outcome measures: (1) severity of disease, (2) duration of symptoms ≥10 days, (3) 90-day recurrence, and (4) 30-day mortality. Covariates including demographic variables, the presence of PCR ribotype 027/NAP/BI1, and potential confounders (immunosuppressive therapy, renal disease, and diabetes; score on Charlson comorbidity index; and time delay between sample testing positive and recruitment) were individually assessed. Severity of disease was assessed both as a CDI outcome and as a baseline predictor for the other outcome measures. Statistically significant covariates were added to the final regression model to produce adjusted *P* values, odds ratios (ORs), and 95% confidence intervals (CIs). All analyses were carried out using SPSS (version 20).

Power calculations were simulated using nQuery Advisor + nTerim (version 2.0). This showed that the power a posteriori was ≥99% for the majority of analyses. However, for analysis of 30-day mortality and disease severity at baseline, power was lower (67% and 75%, respectively; Supplementary Table 2).

## RESULTS

### Patient Demographics

CDI cases and AAD controls were demographically comparable (Table 1). However, mortality at 1 year (35% vs 18%; *P* < .001) and duration of diarrhea symptoms (≥10 days 60% vs 24%; *P* < .0001) were significantly greater among CDI cases. In relation to medication history, 9% (28/308) and 1% (2/145) of CDI cases and AAD controls had prior exposure to PPIs but not antibiotics within 90 days of the development of CDI, respectively, with 58% (180/308) and 54% (79/145) exposed to both an antibiotic and a PPI. Of CDI cases, 41% (127/308) had severe disease and 38% (83/220) experienced recurrence within 90 days. Twenty-eight CDI cases, who had not experienced any recurrence of symptoms but died within the 90-day follow-up period, could not be included in our analysis of recurrence.

**Table 1.** Demographics of Patients With *Clostridium difficile* Infection and Antibiotic-Associated Diarrhea

Patient's Characteristics	CDI Cases (n = 308)	AAD Controls (n = 145)
Female sex	177/308 (57)	81/142 (57)
Age, y, mean (SD)	70.1 (16.4)	65.0 (17.6)
BMI, kg/m^2^, mean (SD)	24.6 (6.8)	26.9 (6.9)
Presence of immunosuppression	52/307 (17)	35/144 (24)
Presence of renal comorbidity	157/307 (51)	82/144 (57)
Presence of diabetes	58/307 (19)	39/144 (27)
Charlson comorbidity score, median (IQR)	1.0 (0.0–2.0)	1.0 (0.0–2.0)
Time delay (testing/recruitment), median (IQR)	3.0 (2.0–4.0)	2.0 (2.0–3.0)
Clinical parameters		
Duration of symptoms ≥10 d	175/290 (60)^a^	32/134 (24)
All-cause mortality within 30 d	26/305 (9)	5/142 (4)
All-cause mortality within 1 y	95/271 (35)^b^	25/141 (18)
Disease severity at baseline	127/308 (41)	…
Recurrence within 90 d	83/220 (38)	…

Data are presented as No. (%) unless otherwise specified. Differences between case and control groups were found to be statistically significant: ^a^ *P* < .0001; ^b^ *P* < .001.

Abbreviations: AAD, antibiotic-associated diarrhea; BMI, body mass index; CDI, *Clostridium difficile* infection; IQR, interquartile range; SD, standard deviation.

### Relationship of Genotype With Serum MBL Concentrations

Of the 9 variants typed in the CDI cases and AAD controls, 3 were excluded: 1 SNP (rs7084554) deviated from Hardy-Weinberg equilibrium (<0.001); rs11003123 was deemed redundant due to complete LD with the Ins/Del polymorphism (rs10556764); and rs36014597 was also in complete LD with both rs10556764 and rs11003123. Of the 6 polymorphisms analyzed, the genotyping success rate was ≥95%. Their minor allele frequencies were in line with those reported in the literature (Supplementary Table 3). For both groups, 7 common haplotypes were derived from the 6 polymorphisms (Supplementary Figure 1), which is consistent with other previous studies in white populations (Table 2) [9, 34].

**Table 2.** Mannose-Binding Lectin (MBL) Serum Concentrations Across *MBL2* Haplotypes in Patients With *Clostridium difficile* Infection and Antibiotic-Associated Diarrhea

Haplotype	*HYPA*	*LYPA*	*LYQA*	*LXPA*	*HYPD*	*LYPB*	*LYQC*
Presence of haplotype							
No. (% frequency)	213 (29)	44 (6)	143 (19)	170 (23)	55 (7)	108 (15)	11 (1)
Median, ng/mL (range)	612 (17–3981)	587 (0–2500)	529 (0–3981)	428 (0–2968)	157 (0–815)	73 (0–637)	48 (0–492)
Absence of haplotype							
No. (% frequency)	198 (9)	367 (17)	268 (13)	241 (11)	356 (17)	303 (14)	400 (19)
Median: absence, ng/mL (range)	171 (0–2374)	388 (0–3981)	324 (0–2968)	377 (0–3981)	484 (0–3981)	568 (0–3981)	420 (0–3981)
*P* value^a^	<.001	.04	<.001	.34	<.001	<.001	.001

^a^ *P* values were calculated using a Mann–Whitney test comparing mannose-binding lectin serum concentrations against the presence/absence of each individual haplotype.

Presence of the mutant allele for all individual *MBL2* variants had a significant influence on serum MBL concentration across all patients, except for the *X* allele encoded by rs7096206 (*P* = .30; Supplementary Table 3). All the assembled *MBL2* haplotypes also significantly impacted on serum concentrations, except for haplotype *LXPA* where there was no difference compared with the overall median value (*P* = .34; Table 2). Genotypic and haplotypic analyses demonstrated that the presence of a variant allele for any of the 3 exonic variants (rs1800451, rs1800450, and rs5030737) were the major contributing factors for lower MBL concentrations (Table 2 and Supplementary Table 3).

**Figure 2.**
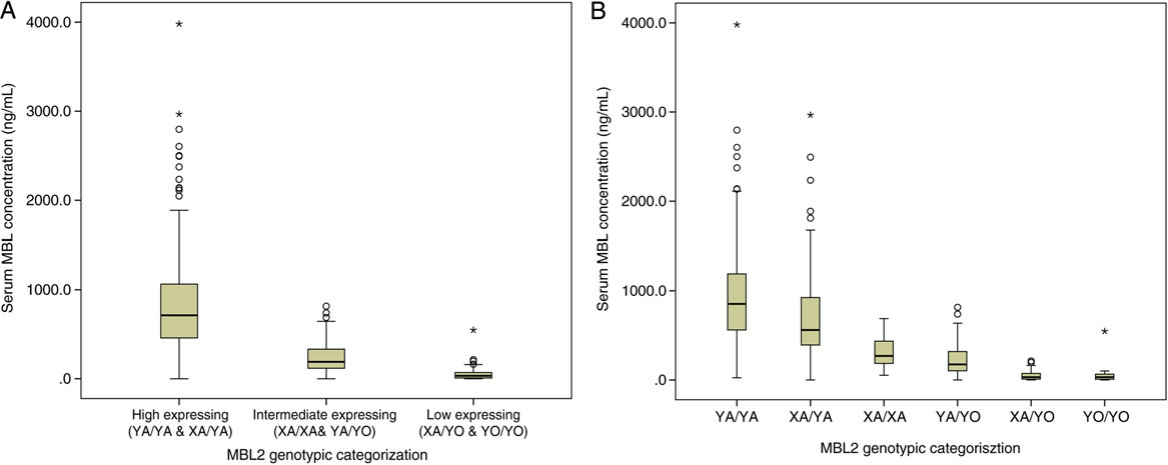
Median serum mannose-binding lectin (MBL) concentrations in relation to 3-tier grouping based on proposed expression profiles (*A*) and individual genotypic groups within proposed expression profiles (*B*). Median serum MBL concentrations were determined across previously defined expression profiles: high (*YA/YA* and *XA/YA*), intermediate (*XA/XA* and *YA/YA*), and low (*XA/YO* and *YO/YO*). Median levels were also determined for the 6 individual genotypic groups across all expression profiles. Abbreviation: MBL, mannose-binding lectin.

Patients with high-expressing genotypes had a median serum MBL concentration of 714 ng/mL, compared with 190 ng/mL with intermediate-expressing genotypes, and 32 ng/mL with low-expressing genotypes (*P* < .001; Table 3; Figure 2*A*). The contribution of the *X* allele, seemingly insignificant when evaluated on an individual basis (Supplementary Table 3), became apparent with a gradual decrease when compared with the equivalent genotypes containing the *Y* allele in the rank order: *XA/YA* < *YA/YA*, *XA/XA* < *XA/YA*, and *XA/YO* < *YA/YO* (Table 3; Figure 2*B*).

**Table 3.** Median Serum Mannose-Binding Lectin Concentrations Across Previously Defined Expression Genotype Groups^a^

MBL Expression Group	Genotype	No.	Median, ng/mL	Combined Median, ng/mL
High	*YA/YA*	124	854	714
	*XA/YA*	113	561	
Intermediate	*XA/XA*	16	270	190
	*YA/YO*	91	175	
Low	*XA/YO*	41	32	32
	*YO/YO*	26	31	

Abbreviation: MBL, mannose-binding lectin.

^a^ Expression groups defined according to Eisen et al [32].

### MBL Deficiency Cutoff Points in Relation to Haplotype Groups

In total 59 (13%), 93 (21%), and 258 (58%) patients had serum MBL concentrations below 50, 100, and 500 ng/mL, respectively. When these data were compared with the “expressing” genotype groups, 78% (42/54) and 68% (59/87) of those with concentrations <50 ng/mL and <100 ng/mL, respectively, were low expressors, compared with 28% (66/236) of those with a concentration <500 ng/mL (Supplementary Table 4). The corresponding figures for high expressors were 4% (2/54), 6% (5/87), and 30% (70/236), respectively. Similarly, 96% (52/54) and 93% (81/87) of those with concentrations <50 ng/mL and <100 ng/mL, respectively, carried the deficient (*O*) haplotypes, compared with 65% (153/236) of those with a concentration <500 ng/mL (Supplementary Table 4). Based on the results above, only the 50 and 100 ng/mL cutoffs were taken forward for further analysis, which is consistent with previous literature [30, 31].

### Comparison of MBL Levels Versus CDI Disease Outcomes

Serum MBL concentrations are shown in Supplementary Table 5. Analysis using both <50 ng/mL and <100 ng/mL as cutoff points to signify deficiency identified no significant differences between CDI cases and AAD controls (*P* = .79 and *P* = .09, respectively) (Table 4). Evaluation of the clinical outcomes in CDI cases showed a significant association with CDI recurrence (*P* < .01 for both; Table 4) with ORs of 3.18 and 2.61 at the <50 ng/mL and <100 ng/mL cutoff points, respectively. No association was identified with any of the other outcomes including prolonged symptoms, 30-day mortality, and disease severity at baseline (Table 4). Despite the strong correlation observed between genotypes/haplotypes and serum MBL concentrations in this cohort, no significant associations were identified between high-, intermediate-, and low-expressing genotypes and CDI disease outcomes (Supplementary Table 6).

**Table 4.** Analysis of *Clostridium difficile* Infection Disease Outcomes Versus Serum Mannose-Binding Lectin Concentration Based on Deficiency Cutoffs of 50 and 100 ng/mL

Concentration	Cases (n = 308)	Controls (n = 145)	*P* Value	OR (95% CI)
<50 ng/mL	41 (13%)	18 (12%)	.79^a^	1.09 (.58–2.06)
<100 ng/mL	70 (23%)	23 (16%)	.09^b^	1.61 (.93–2.79)
	Death (n = 26)	Survival (n = 276)		
<50 ng/mL	3 (12%)	37 (13%)	.78^c^	1.22 (.31–4.82)
<100 ng/mL	5 (19%)	64 (23%)	.84^c^	0.88 (.27–2.89)
	≥10 d (n = 174)	<10 d (n = 113)		
<50 ng/mL	27 (16%)	10 (9%)	.10^d^	1.89 (.88–4.08)
<100 ng/mL	42 (24%)	22 (20%)	.35^d^	1.32 (.74–2.35)
	Recurrence (n = 81)	Nonrecurrence (n = 136)		
<50 ng/mL	18 (22%)	13 (10%)	<.01^e^	3.18 (1.40–7.24)
<100 ng/mL	29 (36%)	24 (18%)	<.01^e^	2.61 (1.35–5.04)
	Severe (n = 125)	Nonsevere (n = 180)		
<50 ng/mL	16 (13%)	25 (14%)	.78^d^	0.91 (.46–1.79)
<100 ng/mL	29 (23%)	41 (23%)	.93^d^	1.02 (.60–1.76)

Data regarding duration of symptoms and disease recurrence was unavailable for 18 and 60 of our cases, respectively. For disease recurrence, an additional 28 patients had died within the follow-up period prior to experiencing any recurrent symptoms and therefore could not be included in the analysis. Serum mannose binding lectin level was unavailable for an additional 3 individuals who were therefore excluded from analysis across all outcomes.

*P* values and ORs were calculated using univariate logistic regression and adjusted for the presence of significant covariates.

Abbreviations: CI, confidence interval; OR, odds ratio.

^a^ Age, body mass index (BMI), time delay between testing positive and recruitment, and the presence of diabetes.

^b^ Age, BMI, time delay between testing positive and recruitment, and the presence of diabetes and immunosuppressive therapy.

^c^ Age, BMI, score on Charlson comorbidity index, and disease severity at baseline.

^d^ No covariates were found to be significant and therefore *P* value remains unadjusted.

^e^ Age.

There was an inverse correlation between MBL and CRP serum concentrations (*R*^2^ = −0.16, *P* = .001; Supplementary Figure 2). No significant correlation was identified with white cell count (*R*^2^ = −0.04, *P* = .44).

## DISCUSSION

Studies evaluating the role of MBL in infectious and immune diseases have focused on either genotype, phenotype, or occasionally on both parameters. The latter approach is preferred as it can show discordance between genotype and phenotype. This study is one of the larger disease-related studies concurrently investigating both genotypic/haplotypic variants and serum concentrations in a white population (Supplementary Table 1) and is the first to demonstrate an association between serum MBL concentrations, but not genotype, and recurrence of CDI within 90 days using two distinct cutoff values for MBL deficiency.

The mechanistic basis of the association is unclear. With other bacterial and viral infections, MBL is thought to be capable of binding to the cell surfaces of invasive pathogens, thereby stimulating a downstream immune response. However, this does not seem to be the case with *C. difficile*, where binding of MBL has been shown to be low [24]. This suggests that MBL deficiency does not per se predispose to CDI and is consistent with the observed lack of difference in circulating concentrations of MBL between CDI cases and AAD controls. MBL has other functions including modulation of inflammation and clearance of apoptotic cells [35]. The former may be relevant to CDI, where MBL may be acting as a modulator of the disease. Consistent with this, clinical manifestations of MBL deficiency appear to be of more relevance either in infants when the immune system is still maturing or in susceptible groups when there is an associated immunodeficiency [36], such as in hospitalized elderly patients or following major clinical interventions. However, these are hypotheses that need further investigation.

Although MBL concentrations remain relatively constant in individuals due to genetic determinants, MBL is known to be a relatively modest acute phase reactant [37]. This is in sharp contrast to other acute phase proteins such as CRP whose concentrations can increase sharply by 10- to 1000-fold during acute inflammation [38]. Elevated CRP concentrations have previously been shown to be associated with various CDI outcomes including disease severity and recurrence [25, 39]. Consistent with this, low MBL concentrations have been associated with an increase in the level of CRP [40], and with our findings of the association with CDI recurrence and inverse correlation with CRP. In keeping with the immunomodulatory effect of MBL, it is known that low concentrations lead to increased secretion of the proinflammatory cytokines interleukin 6, interleukin 1β, and tumor necrosis factor α [40, 41], all of which have also been shown to be elevated in response to CDI [42, 43].

The genetic architecture of the *MBL2* gene is complex (Figure 1), with the existence of numerous common functional polymorphisms and haplotypes (Figure 1; Tables 2 and ;3; and Supplementary Table 3). *MBL2* haplotype frequencies and the corresponding impact on serum MBL concentrations were in line with those previously reported [9, 13] (Table 2). This was also evident after stratification of MBL haplotypes based on previously defined expression genotypes [32, 33], with carriers of low-expressing genotypes showing much lower serum MBL concentrations than both intermediate- and high-expressing genotypes (32 ng/mL vs 190 ng/mL and 714 ng/mL, respectively; Table 3). Despite the strong association observed between *MBL2* genotypes and serum MBL concentrations, and the association between MBL concentrations and CDI recurrence, there was no association between *MBL* genotype and CDI outcomes. Other studies have also identified associations with protein levels, but not with genotype (Supplementary Table 1), highlighting the need to evaluate both MBL genotype and phenotype in infection and other immune conditions. The lack of association between MBL genotype and disease outcome may be due to the incomplete genetic penetrance of *MBL* genetic variation on phenotype. In this study, only 78% and 68% of the low-expressing genotypes accounted for deficient serum levels using the cutoff values of <50 ng/mL and <100 ng/mL, respectively (Supplementary Table 4). Genetic heterogeneity due to functionally related genes such as L-ficolin, *MASP2*, and surfactant proteins may also play a role, but this needs further investigation.

Our study sought to adhere to a stringent methodology through the use of a relatively large cohort size and extensive quality control, but it is not without its limitations. Although there is less chance of MBL concentrations being confounded by infection-related events compared with other response markers, one of the clear drawbacks of this work is the lack of longitudinal measurements, which is now being addressed in a new prospective study. The effect of proteins functionally related to MBL and other markers of inflammation and the relative roles they play in disease modulation need further investigation. Previous studies have used various definitions for MBL deficiency, with commonly used cutoffs ranging from 50 ng/mL [30] to 500 ng/mL [32]. It is thus difficult to compare results across different study groups given the heterogeneity of platforms, profile of cohorts, and standards adopted for the measurement of MBL. Discrepancies between studies could be due to low sample sizes, poor assay performance, and differences in techniques adopted by laboratories. We have tried to overcome some of these limitations by evaluating a number of cutoff levels, but there is a need for international consensus and harmonization in this area.

In conclusion, our data suggest that low serum MBL concentrations may act as a predictor of CDI recurrence. Further work is needed to validate these findings in an independent cohort of patients and to evaluate the mechanistic basis of this association. This area of research would also be advanced through consensus on definitions of deficiency, standardization of methods employed for measurement of serum concentrations, and further evaluation of the genotype–phenotype relationships.

## Supplementary Data

Supplementary materials are available at *Clinical Infectious Diseases* online (http://cid.oxfordjournals.org). Supplementary materials consist of data provided by the author that are published to benefit the reader. The posted materials are not copyedited. The contents of all supplementary data are the sole responsibility of the authors. Questions or messages regarding errors should be addressed to the author.

Supplementary Data

## References

[1] Turner MW (2003). The role of mannose-binding lectin in health and disease. Mol Immunol.

[2] Super M, Thiel S, Lu J, Levinsky RJ, Turner MW (1989). Association of low levels of mannan-binding protein with a common defect of opsonisation. Lancet.

[3] Wang M, Chen Y, Zhang Y, Zhang L, Lu X, Chen Z (2011). Mannan-binding lectin directly interacts with Toll-like receptor 4 and suppresses lipopolysaccharide-induced inflammatory cytokine secretion from THP-1 cells. Cell Mol Immunol.

[4] Møller-Kristensen M, Ip WK, Shi L (2006). Deficiency of mannose-binding lectin greatly increases susceptibility to postburn infection with *Pseudomonas aeruginosa*. J Immunol.

[5] Shi L, Takahashi K, Dundee J (2004). Mannose-binding lectin-deficient mice are susceptible to infection with *Staphylococcus aureus*. J Exp Med.

[6] Jack DL, Klein NJ, Turner MW (2001). Mannose-binding lectin: targeting the microbial world for complement attack and opsonophagocytosis. Immunol Rev.

[7] Osthoff M, Trendelenburg M (2013). Impact of mannose-binding lectin deficiency on radiocontrast-induced renal dysfunction. Biomed Res Int.

[8] Madsen HO, Satz ML, Hogh B, Svejgaard A, Garred P (1998). Different molecular events result in low protein levels of mannan-binding lectin in populations from southeast Africa and South America. J Immunol.

[9] Steffensen R, Thiel S, Varming K, Jersild C, Jensenius JC (2000). Detection of structural gene mutations and promoter polymorphisms in the mannan-binding lectin (MBL) gene by polymerase chain reaction with sequence-specific primers. J Immunol Methods.

[10] Harrison E, Singh A, Morris J (2012). Mannose-binding lectin genotype and serum levels in patients with chronic and allergic pulmonary aspergillosis. Int J Immunogenet.

[11] Guo N, Mogues T, Weremowicz S, Morton CC, Sastry KN (1998). The human ortholog of rhesus mannose-binding protein-A gene is an expressed pseudogene that localizes to chromosome 10. Mamm Genome.

[12] Sastry R, Wang JS, Brown DC, Ezekowitz RA, Tauber AI, Sastry KN (1995). Characterization of murine mannose-binding protein genes Mbl1 and Mbl2 reveals features common to other collectin genes. Mamm Genome.

[13] Madsen HO, Garred P, Thiel S (1995). Interplay between promoter and structural gene variants control basal serum level of mannan-binding protein. J Immunol.

[14] Garred P, Larsen F, Seyfarth J, Fujita R, Madsen HO (2006). Mannose-binding lectin and its genetic variants. Genes Immun.

[15] Bernig T, Taylor JG, Foster CB, Staats B, Yeager M, Chanock SJ (2004). Sequence analysis of the mannose-binding lectin (MBL2) gene reveals a high degree of heterozygosity with evidence of selection. Genes Immun.

[16] El Feghaly RE, Stauber JL, Deych E, Gonzalez C, Tarr PI, Haslam DB (2013). Markers of intestinal inflammation, not bacterial burden, correlate with clinical outcomes in *Clostridium difficile* infection. Clin Infect Dis.

[17] Shastri YM, Bergis D, Povse N (2008). Prospective multicenter study evaluating fecal calprotectin in adult acute bacterial diarrhea. Am J Med.

[18] Rao K, Erb-Downward JR, Walk ST (2014). The systemic inflammatory response to *Clostridium difficile* infection. PLoS One.

[19] Buonomo EL, Madan R, Pramoonjago P, Li L, Okusa MD, Petri WA (2013). Role of interleukin 23 signaling in *Clostridium difficile* colitis. J Infect Dis.

[20] Rao K, Walk ST, Micic D (2013). Procalcitonin levels associate with severity of *Clostridium difficile* infection. PLoS One.

[21] Hu MY, Katchar K, Kyne L (2009). Prospective derivation and validation of a clinical prediction rule for recurrent *Clostridium difficile* infection. Gastroenterology.

[22] Hensgens MP, Dekkers OM, Goorhuis A, LeCessie S, Kuijper EJ (2014). Predicting a complicated course of *Clostridium difficile* infection at the bedside. Clin Microbiol Infect.

[23] Butt E, Foster JA, Keedwell E (2013). Derivation and validation of a simple, accurate and robust prediction rule for risk of mortality in patients with *Clostridium difficile* infection. BMC Infect Dis.

[24] Townsend R, Read RC, Turner MW, Klein NJ, Jack DL (2001). Differential recognition of obligate anaerobic bacteria by human mannose-binding lectin. Clin Exp Immunol.

[25] Eyre DW, Walker AS, Wyllie D (2012). Predictors of first recurrence of *Clostridium difficile* infection: implications for initial management. Clin Infect Dis.

[26] Ryan A, Lynch M, Smith SM (2011). A role for TLR4 in *Clostridium difficile* infection and the recognition of surface layer proteins. PLoS Pathog.

[27] Miyajima F, Swale A, Zhang JE (2014). Is the interleukin 8 promoter polymorphism rs4073/-251T >A associated with *Clostridium difficile* infection?. Clin Infect Dis.

[28] Miyajima F, Roberts P, Swale A (2011). Characterisation and carriage ratio of *Clostridium difficile* strains isolated from a community-dwelling elderly population in the United Kingdom. PLoS One.

[29] Public Health England (2013). Updated guidance on the management and treatment of *Clostridium difficile* infection. http://www.hpa.org.uk/webc/HPAwebFile/HPAweb_C/1317138914904.

[30] Gröndahl-Yli-Hannuksela K, Viander M, Mertsola J, He Q (2013). Increased risk of pertussis in adult patients with mannose-binding lectin deficiency. APMIS.

[31] Seibold F, Konrad A, Flogerzi B (2004). Genetic variants of the mannan-binding lectin are associated with immune reactivity to mannans in Crohn's disease. Gastroenterology.

[32] Eisen DP, Dean MM, Boermeester MA (2008). Low serum mannose-binding lectin level increases the risk of death due to pneumococcal infection. Clin Infect Dis.

[33] Chalmers JD, McHugh BJ, Doherty C (2013). Mannose-binding lectin deficiency and disease severity in non-cystic fibrosis bronchiectasis: a prospective study. Lancet Respir Med.

[34] Adamek M, Heyder J, Heinold A, Fiedler G, Opelz G, Tran TH (2013). Characterization of mannose-binding lectin (MBL) variants by allele-specific sequencing of MBL2 and determination of serum MBL protein levels. Tissue Antigens.

[35] Dommett RM, Klein N, Turner MW (2006). Mannose-binding lectin in innate immunity: past, present and future. Tissue Antigens.

[36] Koch A, Melbye M, Sørensen P (2001). Acute respiratory tract infections and mannose-binding lectin insufficiency during early childhood. JAMA.

[37] Dean MM, Minchinton RM, Heatley S, Eisen DP (2005). Mannose binding lectin acute phase activity in patients with severe infection. J Clin Immunol.

[38] Ip WK, Takahashi K, Ezekowitz RA, Stuart LM (2009). Mannose-binding lectin and innate immunity. Immunol Rev.

[39] Khanafer N, Touré A, Chambrier C (2013). Predictors of *Clostridium difficile* infection severity in patients hospitalised in medical intensive care. World J Gastroenterol.

[40] Garred P, Pressler T, Lanng S (2002). Mannose-binding lectin (MBL) therapy in an MBL-deficient patient with severe cystic fibrosis lung disease. Pediatr Pulmonol.

[41] Jack DL, Read RC, Tenner AJ, Frosch M, Turner MW, Klein NJ (2001). Mannose-binding lectin regulates the inflammatory response of human professional phagocytes to *Neisseria meningitidis* serogroup B. J Infect Dis.

[42] Hirota SA, Iablokov V, Tulk SE (2012). Intrarectal instillation of *Clostridium difficile* toxin A triggers colonic inflammation and tissue damage: development of a novel and efficient mouse model of *Clostridium difficile* toxin exposure. Infect Immun.

[43] Vohra P, Poxton IR (2012). Induction of cytokines in a macrophage cell line by proteins of *Clostridium difficile*. FEMS Immunol Med Microbiol.

