# Supplementary material for: Serum Mannose-Binding Lectin Concentration, but Not Genotype, Is Associated With Clostridium difficile Infection Recurrence: A Prospective Cohort Study
Source: Clin Infect Dis. 2014 Aug 28;59(10):1429–36. doi: 10.1093/cid/ciu666 (PMC4207421; doi:10.1093/cid/ciu666)
Supplement: Supplementary Data [file supp_59_10_1429__index.html]

Serum mannose-binding lectin concentration, but not genotype, is associated with Clostridium difficile infection recurrence: a prospective cohort study — Serum Mannose-Binding Lectin Concentration, but Not Genotype, Is Associated With Clostridium difficile Infection Recurrence: A Prospective Cohort Study — Serum Mannose-Binding Lectin Concentration, but Not Genotype, Is Associated With Clostridium difficile Infection Recurrence: A Prospective Cohort Study — Supplementary Data 

# Serum Mannose-Binding Lectin Concentration, but Not Genotype, Is Associated With *Clostridium difficile* Infection Recurrence: A Prospective Cohort Study

## Supplementary Data

Supplementary Data

**Files in this Data Supplement:**

- Supplementary Figure 1 - pdf file
- Supplementary Figure 2 - pdf file
- Supplementary Tables - docx file
