# Supplementary material for: Serum Mannose-Binding Lectin Concentration, but Not Genotype, Is Associated With Clostridium difficile Infection Recurrence: A Prospective Cohort Study
Source: Clin Infect Dis. 2014 Aug 28;59(10):1429–36. doi: 10.1093/cid/ciu666 (PMC4207421; doi:10.1093/cid/ciu666)

Supplementary Figure 1 – Linkage disequilibrium plots, detailing D' (A) and R^2 (B), for the 6 *MBL2* polymorphisms known to affect protein expression levels. Here, P/Q refers to the 6 bp deletion (rs10556764) used as a proxy SNP in this study

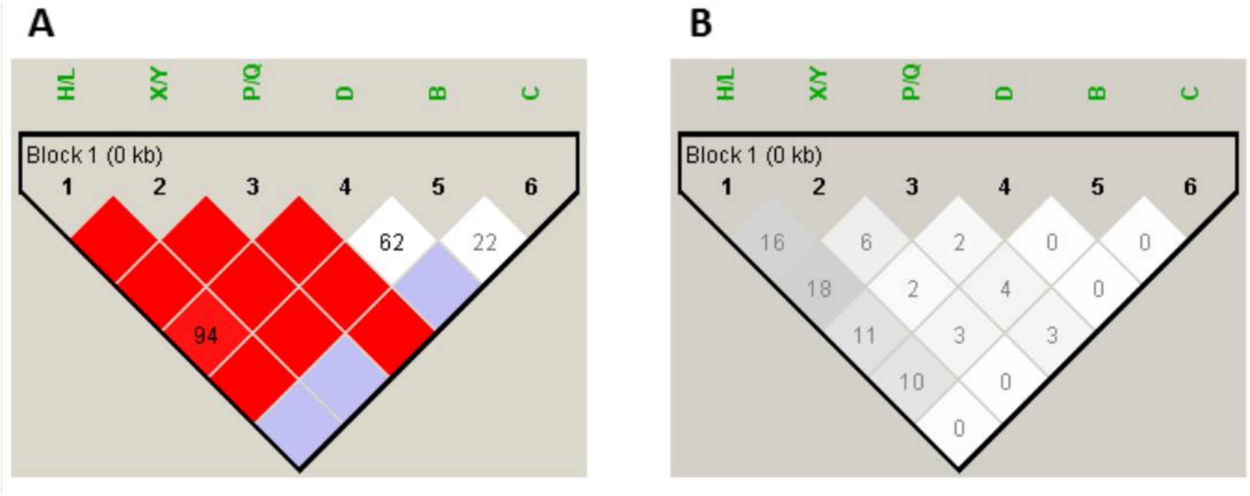

Supplement: Supplementary Data [file supp_ciu666_ciu666supp_fig1.pdf]
