# Supplementary material for: Serum Mannose-Binding Lectin Concentration, but Not Genotype, Is Associated With Clostridium difficile Infection Recurrence: A Prospective Cohort Study
Source: Clin Infect Dis. 2014 Aug 28;59(10):1429–36. doi: 10.1093/cid/ciu666 (PMC4207421; doi:10.1093/cid/ciu666)

Supplementary Figure 2 – Correlation plots comparing MBL concentrations against routine blood markers (A) CRP and (B) White blood cells

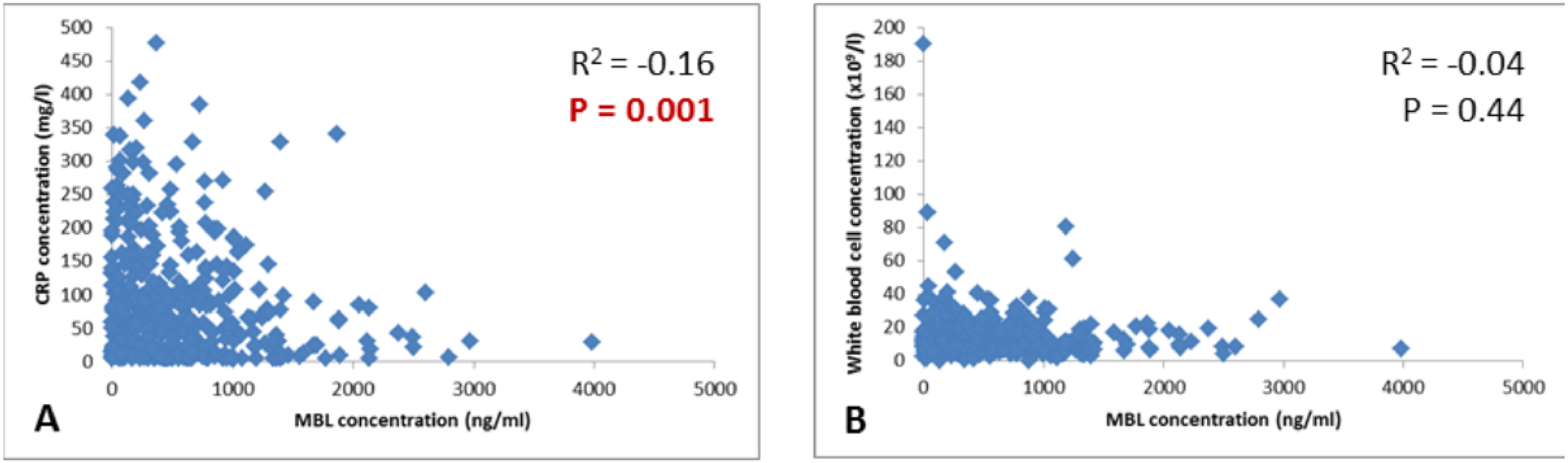

Supplement: Supplementary Data [file supp_ciu666_ciu666supp_fig2.pdf]
