# Supplementary material for: Serum Mannose-Binding Lectin Concentration, but Not Genotype, Is Associated With Clostridium difficile Infection Recurrence: A Prospective Cohort Study
Source: Clin Infect Dis. 2014 Aug 28;59(10):1429–36. doi: 10.1093/cid/ciu666 (PMC4207421; doi:10.1093/cid/ciu666)
Supplement: Supplementary Data [file supp_ciu666_ciu666supp_tables.docx]

**Supplementary Table 1 – Disease-related studies investigating both *MBL2* genotypic data and protein concentrations of MBL in White adult population**

| **Study** | **Country** | **Disease** | **n*** | **Association with outcome** |
| --- | --- | --- | --- | --- |
| **Infection-related conditions** | | | | |
| Garred et al. 1997 [1] | Denmark | HIV | 96 | Yes (G) |
| Garred et al. 1999 [2] | Denmark | Infection in SLE patients | 91 | Yes (G) |
| Soborg et al. 2003 [3] | Denmark | Tuberculosis | 59 | Yes (G) |
| Yang et al. 2003 [4] | Australia | Chronic obstructive pulmonary disease | 82 | Yes (G) |
| Bouwman et al. 2005 [5] | Netherlands | Severe infection post-liver transplant | 49 | Yes (G) |
| Druszczyńska et al. 2006 [6] | Poland | Tuberculosis | 108 | Yes (P) |
| Eisen et al. 2006 [7] | Australia | Sepsis | 170 | Yes (G+P) |
| Gordon et al. 2006 [8] | United Kingdom | Sepsis | 80 | Yes (G+P) |
| Perez-Castellano et al. 2006 [9] | Spain | Community-acquired pneumonia | 97 | No |
| Louropoulou et al. 2008 [10] | Netherlands | Periodontitis | 92 | No |
| Van Till et al. 2008 [11] | Netherlands | AYI in secondary peritonitis patients | 88 | Yes (G+P) |
| Ampel et al. 2009 [12] | USA | Coccidioidomyosis | 38 | Yes (P) |
| Harrison et al. 2012 [13] | United Kingdom | Aspergillosis | 108 | Yes (G+P) |
| Navratilova et al. 2012 [14] | Czech Republic | Prosthetic Joint Infection | 92 | Yes (G) |
| Wong et al. 2012 [15] | Sweden | Infection in neutropaenic chemotherapy patients | 108 | No |
| Chalmers et al. 2013 [16] | United Kingdom | Infection in bronchiecstasis patients | 470 | Yes (G+P) |
| Osthoff et al. 2013 [17] | Australia | *Staphylococcus Aureus* infection | 70 | Yes (G+P) |
| **Other conditions** | | | | |
| Garred et al. 2000 [18] | Denmark | Rheumatoid arthritis | 189 | Yes (G) |
| Megia et al. 2004 [19] | Spain | Gestational diabetes mellitus | 105 | Yes (G+P) |
| Seibold et al. 2004 [20] | Switzerland | Inflammatory bowel disease | 76 | Yes (G+P) |
| Kamesh et al. 2007 [21] | United Kingdom | ANCA-associated vessel vasculitis | 137 | No |
| Nielsen et al. 2007 [22] | Denmark | Crohn’s disease | 171 | No |
| Swierzko et al. 2007 [23] | Poland | Reproductive tumours | 183 | No |
| Christiansen et al. 2009 [24] | Denmark | Recurrent late pregnancy loss | 75 | Yes (G) |
| Kaunisto et al. 2009 [25] | Finland | Diabetes | 1064 | No |
| Hoffmann et al. 2010 [26] | Germany | Inflammatory bowel disease | 181 | No |
| Troelsen et al. 2010 [27] | Denmark | SLE | 41 | Yes (G+P) |
| Troelsen et al. 2010 [28] | Denmark | Rheumatoid arthritis | 114 | Yes (P) |
| Troelsen et al. 2010 [29] | Denmark | Rheumatoid arthritis | 229 | Yes (G+P) |
| Kiseljaković et al. 2014 [30] | Bosnia-Herzegovina | Postmenopausal osteoporosis | 37 | No |

*n: number; HIV: Human Immunodeficiency virus; SLE: Systemic lupus erythematosus; AYI: Abdominal yeast infection; ANCA: Anti-neutrophil cytoplasmic antibody; G: Genetic; P: protein*

**This refers to total number of White patients differs with both serum concentration & genotypic data and therefore may differ from total study number*

**Supplementary Table 2 - Assessment of power across clinical outcome associated with *Clostridium difficile* infection**

| **Disease outcome** | **Number** | **Percentage power achieved** |
| --- | --- | --- |
| Case versus control | 305 vs. 142 | 99 |
| 30-day mortality | 26 vs. 276 | 67^a^ |
| Prolonged symptoms | 175 vs. 115 | 99 |
| 90-day recurrence | 83 vs. 137 | 99 |
| Disease severity at baseline | 126 vs. 180 | 75^b^ |

*a: Achieving 80% power would require 66 patients in both sample groups*

*b: Achieving 80% power would require 166 patients in both sample groups*

**Supplementary Table 3 – Overview of the six *MBL2* variants employed for the haplotype construction and association with MBL concentrations**

|  | **rs11003125** | **rs10556764** | **rs7096206** | **rs5030737** | **rs1800450** | **rs1800451** |
| --- | --- | --- | --- | --- | --- | --- |
| **Nucleotide change** | -550  G>C | -327 to -332  CTCTTT/- | -221  G>C | +219  G>A | +227  C>T | +235  C>T |
| **Haplotype component** | *H/L* | Ins/Del  (*P/Q* proxy) | *X/Y* | Codon 52 (*D*) | Codon 54 (*B*) | Codon 57 (*C*) |
| **Minor allele** | *H* | Del | *Y* | *D* | *B* | *C* |
| **MAF** | 0.36 | 0.20 | 0.23 | 0.07 | 0.14 | 0.01 |
| **Median for the presence of minor variant: ng/ml (n)** | 537.5 (262) | 503.2 (158) | 396.1 (185) | 158.3 (58) | 73.8 (113) | 51.0 (12) |
| **Median for the absence of minor variant: ng/ml (n)** | 223.1 (180) | 330.6 (265) | 376.8 (256) | 483.8 (373) | 578.5 (315) | 419.7 (419) |
| **P-value*** | **<0.001** | **<0.001** | 0.30 | **<0.001** | **<0.001** | **<0.001** |

*MAF = Minor Allele Frequency; n: Number; *P-value was calculated using a Kruskal-Wallis test comparing median concentrations for presence versus absence of the minor variant of each individual SNP, across all patients (cases and controls combined)*

**Supplementary Table 4 – Distribution of expression genotypes and deficiency haplotypes across three different serum MBL deficiency cut-offs***

| Cut-off (ng/ml) | n (%) | | |  |
| --- | --- | --- | --- | --- |
|  | High  expressors | Intermediate expressors | Low  expressors |  |
| 50 (n=54) | 2 (4) | 10 (18) | 42 (78) |  |
| 100 (n=87) | 5 (6) | 23 (26) | 59 (68) |  |
| 500 (n=236) | 70 (30) | 100 (42) | 66 (28) |  |
|  | Homozygous non-deficient haplotypes | Heterozygous deficient haplotypes | Homozygous deficient haplotypes | Dominant model^a^ |
| 50 (n=54) | 2 (4) | 34 (63) | 18 (33) | 52 (96) |
| 100 (n=87) | 6 (7) | 57 (65) | 24 (28) | 81 (93) |
| 500 (n=236) | 83 (35) | 128 (54) | 25 (11) | 153 (65) |

*n: number; ^a^ Dominant model refers to the presence of ≥1 deficiency haplotype across both the maternal and paternal haplotypes of each patient*

**The number of patients deemed high (YA/YA & XA/YA), intermediate (XA/XA & YA/YO) and low (XA/YO & YO/YO) expressors, plus the number of patients carrying either deficient (O) or non-deficient (A) haplotypes, was assessed across three individual serum MBL deficiency cut-offs.*

**Supplementary Table 5 - Descriptive MBL serum concentrations in relation to *Clostridium difficile* infection disease outcomes**

|  | N (% missing) | Median, ng/ml (IQR) | Min. – Max. |
| --- | --- | --- | --- |
| Case versus Control | | | |
| Case (n=308) | 305 (1.0) | 361.8 (128.3-747.7) | 0.0-3,980.8 |
| Control (n=145) | 141 (2.8) | 491.9 (160.0-856.0) | 0.0-2,493.4 |
| Death within 30 days | | | |
| Death (n=26) | 26 (0.0) | 330.3 (115.9-673.0) | 4.1-1,378.1 |
| Non-death (n=279) | 276 (1.1) | 372.9 (128.4-754.2) | 0.0-3,980.8 |
| Duration ≥10 days | | | |
| Yes (n=175) | 174 (0.6) | 332.4 (113.4-698.7) | 0.0-2,604.2 |
| No (n=115) | 113 (1.7) | 425.5 (163.9-896.5) | 0.0-3,980.8 |
| Recurrence within 90 days | | | |
| Recurrence (n=83) | 81 (2.4) | 196.7 (60.9-570.4) | 0.0-2,134.5 |
| Non-recurrence (n=137) | 136 (0.7) | 452.1 (169.6-844.5) | 0.0-3,980.8 |
| Severity at baseline | | | |
| Severe (n=127) | 125 (1.9) | 372.1 (128.3-728.2) | 0.0-2,967.5 |
| Non-severe (n=181) | 180 (0.5) | 354.6 (128.0-787.1) | 0.0-3,980.8 |

*n: number; IQR: interquartile range; Min: minimum; Max: maximum*

**Supplementary Table 6 – Analysis of *Clostridium difficile* infection disease outcomes versus high, intermediate and low expressing *MBL2* genotypes**

|  | Case (n=308) | Control (n=145) | P-value^a^ | OR (95% CI) |
| --- | --- | --- | --- | --- |
| High expressing group (comparator) | 165 | 73 | 0.86 | - |
| Intermediate expressing group | 75 | 34 | 0.75 | 0.92 (0.55-1.55) |
| Low expressing group | 44 | 24 | 0.61 | 0.85 (0.47-1.56) |
|  | Death (n=26) | Survival (n=276) | P-value^b^ | OR (95% CI) |
| High expressing group (comparator) | 14 | 150 | 0.77 | - |
| Intermediate expressing group | 8 | 67 | 0.47 | 1.49 (0.50-4.43) |
| Low expressing group | 3 | 40 | 0.75 | 1.26 (0.31-5.03) |
|  | ≥10 days (n=165) | <10 days (n=103) | P-value^c^ | OR (95% CI) |
| High expressing group (comparator) | 99 | 57 | 0.68 | - |
| Intermediate expressing group | 43 | 28 | 0.68 | 0.88 (0.50-1.57) |
| Low expressing group | 23 | 18 | 0.39 | 0.74 (0.37-1.48) |
|  | Recurrence (n=78) | Non-recurrence (n=133) | P-value^d^ | OR (95% CI) |
| High expressing group (comparator) | 42 | 83 | 0.46 | - |
| Intermediate expressing group | 22 | 31 | 0.32 | 1.43 (0.71-2.86) |
| Low expressing group | 14 | 19 | 0.33 | 1.50 (0.66-3.39) |
|  | Severe (n=120) | Non-severe (n=185) | P-value^c^ | OR (95% CI) |
| High expressing group (comparator) | 64 | 101 | 0.33 |  |
| Intermediate expressing group | 36 | 39 | 0.18 | 1.46 (0.84-2.53) |
| Low expressing group | 16 | 28 | 0.77 | 0.90 (0.45-1.80) |

*n: number; OR: odds ratio; CI: confidence interval;*

*P-values & ORs were calculated using univariate logistic regression and adjusted for the presence of significant covariates: ^a^ Age, BMI, time delay between testing positive and recruitment & the presence of diabetes; ^b^ Age, BMI, score on Charlson Comorbidity Index and disease severity at baseline; ^c^ No covariates were found to be significant & therefore P-value remains unadjusted; ^d^ Age*

**REFERENCES**

1. Garred P, Madsen HO, Balslev U, et al. Susceptibility to HIV infection and progression of AIDS in relation to variant alleles of mannose-binding lectin. Lancet **1997**; 349(9047): 236-40.

2. Garred P, Madsen HO, Halberg P, et al. Mannose-binding lectin polymorphisms and susceptibility to infection in systemic lupus erythematosus. Arthritis Rheum **1999**; 42(10): 2145-52.

3. Søborg C, Madsen HO, Andersen AB, Lillebaek T, Kok-Jensen A, Garred P. Mannose-binding lectin polymorphisms in clinical tuberculosis. J Infect Dis **2003**; 188(5): 777-82.

4. Yang IA, Seeney SL, Wolter JM, et al. Mannose-binding lectin gene polymorphism predicts hospital admissions for COPD infections. Genes Immun **2003**; 4(4): 269-74.

5. Bouwman LH, Roos A, Terpstra OT, et al. Mannose binding lectin gene polymorphisms confer a major risk for severe infections after liver transplantation. Gastroenterology **2005**; 129(2): 408-14.

6. Druszczyńska M, Strapagiel D, Kwiatkowska S, et al. Tuberculosis bacilli still posing a threat. Polymorphism of genes regulating anti-mycobacterial properties of macrophages. Pol J Microbiol **2006**; 55(1): 7-12.

7. Eisen DP, Dean MM, Thomas P, et al. Low mannose-binding lectin function is associated with sepsis in adult patients. FEMS Immunol Med Microbiol **2006**; 48(2): 274-82.

8. Gordon AC, Waheed U, Hansen TK, et al. Mannose-binding lectin polymorphisms in severe sepsis: relationship to levels, incidence, and outcome. Shock **2006**; 25(1): 88-93.

9. Perez-Castellano M, Peñaranda M, Payeras A, et al. Mannose-binding lectin does not act as an acute-phase reactant in adults with community-acquired pneumococcal pneumonia. Clin Exp Immunol **2006**; 145(2): 228-34.

10. Louropoulou A, van der Velden U, Schoenmaker T, Catsburg A, Savelkoul PH, Loos BG. Mannose-binding lectin gene polymorphisms in relation to periodontitis. J Clin Periodontol **2008**; 35(11): 923-30.

11. van Till JW, Modderman PW, de Boer M, Hart MH, Beld MG, Boermeester MA. Mannose-binding lectin deficiency facilitates abdominal Candida infections in patients with secondary peritonitis. Clin Vaccine Immunol **2008**; 15(1): 65-70.

12. Ampel NM, Dionne SO, Giblin A, Podany AB, Galgiani J. Mannose-binding lectin serum levels are low in persons with clinically active coccidioidomycosis. Mycopathologia **2009**; 167(4): 173-80.

13. Harrison E, Singh A, Morris J, et al. Mannose-binding lectin genotype and serum levels in patients with chronic and allergic pulmonary aspergillosis. Int J Immunogenet **2012**; 39(3): 224-32.

14. Navratilova Z, Gallo J, Mrazek F, Lostak J, Petrek M. MBL2 gene variation affecting serum MBL is associated with prosthetic joint infection in Czech patients after total joint arthroplasty. Tissue Antigens **2012**; 80(5): 444-51.

15. Wong M, Öhrmalm L, Broliden K, Aust C, Hibberd M, Tolfvenstam T. Mannose-binding lectin 2 polymorphisms do not influence frequency or type of infection in adults with chemotherapy induced neutropaenia. PLoS One **2012**; 7(2): e30819.

16. Chalmers JD, McHugh BJ, Doherty C, et al. Mannose-binding lectin deficiency and disease severity in non-cystic fibrosis bronchiectasis: a prospective study. Lancet Respir Med **2013**; 1(3): 224-32.

17. Osthoff M, Au Yong HM, Dean MM, Eisen DP. Significance of mannose-binding lectin deficiency and nucleotide-binding oligomerization domain 2 polymorphisms in staphylococcus aureus bloodstream infections: a case-control study. PLoS One **2013**; 8(9): e76218.

18. Garred P, Madsen HO, Marquart H, et al. Two edged role of mannose binding lectin in rheumatoid arthritis: a cross sectional study. J Rheumatol **2000**; 27(1): 26-34.

19. Megia A, Gallart L, Fernández-Real JM, et al. Mannose-binding lectin gene polymorphisms are associated with gestational diabetes mellitus. J Clin Endocrinol Metab **2004**; 89(10): 5081-7.

20. Seibold F, Konrad A, Flogerzi B, et al. Genetic variants of the mannan-binding lectin are associated with immune reactivity to mannans in Crohn's disease. Gastroenterology **2004**; 127(4): 1076-84.

21. Kamesh L, Heward JM, Williams JM, Gough SC, Savage CO, Harper L. Mannose-binding lectin gene polymorphisms in a cohort study of ANCA-associated small vessel vasculitis. Rheumatology (Oxford) **2007**; 46(7): 1076-8.

22. Nielsen RG, Vind I, Munkholm P, et al. Genetic polymorphisms of mannan binding lectin (MBL), serum levels of MBL, the MBL associated serine protease and H-ficolin in patients with Crohn's disease. Gut **2007**; 56(2): 311-2.

23. Swierzko AS, Florczak K, Cedzyński M, et al. Mannan-binding lectin (MBL) in women with tumours of the reproductive system. Cancer Immunol Immunother **2007**; 56(7): 959-71.

24. Christiansen OB, Nielsen HS, Lund M, Steffensen R, Varming K. Mannose-binding lectin-2 genotypes and recurrent late pregnancy losses. Hum Reprod **2009**; 24(2): 291-9.

25. Kaunisto MA, Sjölind L, Sallinen R, et al. Elevated MBL concentrations are not an indication of association between the MBL2 gene and type 1 diabetes or diabetic nephropathy. Diabetes **2009**; 58(7): 1710-4.

26. Hoffmann C, Hoffmann P, Lun A, et al. Is there a role for mannan-binding lectin in the diagnosis of inflammatory bowel disease? Immunogenetics **2010**; 62(4): 231-5.

27. Troelsen LN, Garred P, Christiansen B, Torp-Pedersen C, Jacobsen S. Genetically determined serum levels of mannose-binding lectin correlate negatively with common carotid intima-media thickness in systemic lupus erythematosus. J Rheumatol **2010**; 37(9): 1815-21.

28. Troelsen LN, Garred P, Christiansen B, et al. Double role of mannose-binding lectin in relation to carotid intima-media thickness in patients with rheumatoid arthritis. Mol Immunol **2010**; 47(4): 713-8.

29. Troelsen LN, Garred P, Jacobsen S. Mortality and predictors of mortality in rheumatoid arthritis--a role for mannose-binding lectin? J Rheumatol **2010**; 37(3): 536-43.

30. Kiseljaković E, Hasić S, Valjevac A, et al. Association of mannose-binding lectin 2 (mbl2) gene heterogeneity and its serum concentration with osteoporosis in postmenopausal women. Bosn J Basic Med Sci **2014**; 14(1): 25-9.
